# Supplementary material for: Modeling glioblastoma heterogeneity as a dynamic network of cell states
Source: Mol Syst Biol. 2021 Sep 16;17(9):e10105. doi: 10.15252/msb.202010105 (PMC8444284; doi:10.15252/msb.202010105)
Supplement: Supplementary file 6 — Source Data for Figure 5 [file MSB-17-e10105-s004.zip › Figure5A_sourcedata/GSEA_3071/nosigdb_stateIV.GseaPreranked.1622795164626/RICHARDS_NOWAKOWSKI_SCIENCE_2017_IPC_NEN1_UPREG.html]

Details for gene set RICHARDS\_NOWAKOWSKI\_SCIENCE\_2017\_IPC\_NEN1\_UPREG[GSEA]

|  || Dataset | state53071 |
| Phenotype | NoPhenotypeAvailable |
| Upregulated in class | na\_pos |
| GeneSet | RICHARDS\_NOWAKOWSKI\_SCIENCE\_2017\_IPC\_NEN1\_UPREG |
| Enrichment Score (ES) | 0.50520337 |
| Normalized Enrichment Score (NES) | 3.051827 |
| Nominal p-value | 0.0 |
| FDR q-value | 0.0 |
| FWER p-Value | 0.0 |
Table: GSEA Results Summary

  

Fig 1: Enrichment plot: RICHARDS\_NOWAKOWSKI\_SCIENCE\_2017\_IPC\_NEN1\_UPREG      
 Profile of the Running ES Score & Positions of GeneSet Members on the Rank Ordered List

  

| PROBE | GENE SYMBOL | GENE\_TITLE | RANK IN GENE LIST | RANK METRIC SCORE | RUNNING ES | CORE ENRICHMENT || 1 | NFIA |  |  | 24 | 2.181 | 0.0120 | Yes |
| 2 | LINC00461 |  |  | 30 | 2.010 | 0.0263 | Yes |
| 3 | MEIS2 |  |  | 32 | 2.004 | 0.0414 | Yes |
| 4 | MAP2 |  |  | 55 | 1.811 | 0.0510 | Yes |
| 5 | SOX11 |  |  | 56 | 1.811 | 0.0648 | Yes |
| 6 | FGD4 |  |  | 65 | 1.758 | 0.0767 | Yes |
| 7 | POU3F2 |  |  | 70 | 1.728 | 0.0891 | Yes |
| 8 | TAGLN3 |  |  | 71 | 1.720 | 0.1022 | Yes |
| 9 | ZBTB20 |  |  | 81 | 1.628 | 0.1129 | Yes |
| 10 | MMP16 |  |  | 90 | 1.578 | 0.1234 | Yes |
| 11 | PGAP1 |  |  | 94 | 1.556 | 0.1347 | Yes |
| 12 | TCF4 |  |  | 96 | 1.551 | 0.1463 | Yes |
| 13 | NEDD4L |  |  | 100 | 1.535 | 0.1575 | Yes |
| 14 | ROBO2 |  |  | 114 | 1.499 | 0.1664 | Yes |
| 15 | NFIB |  |  | 117 | 1.491 | 0.1774 | Yes |
| 16 | MYO10 |  |  | 121 | 1.484 | 0.1881 | Yes |
| 17 | GPRC5B |  |  | 142 | 1.390 | 0.1948 | Yes |
| 18 | CHD7 |  |  | 144 | 1.386 | 0.2052 | Yes |
| 19 | SEL1L3 |  |  | 151 | 1.371 | 0.2145 | Yes |
| 20 | NFIX |  |  | 162 | 1.345 | 0.2228 | Yes |
| 21 | ZFHX4 |  |  | 166 | 1.335 | 0.2324 | Yes |
| 22 | PLCB1 |  |  | 168 | 1.323 | 0.2423 | Yes |
| 23 | NKAIN3 |  |  | 183 | 1.295 | 0.2495 | Yes |
| 24 | CUX1 |  |  | 184 | 1.295 | 0.2594 | Yes |
| 25 | MEIS3 |  |  | 205 | 1.253 | 0.2650 | Yes |
| 26 | FBLN1 |  |  | 213 | 1.226 | 0.2730 | Yes |
| 27 | MYEF2 |  |  | 225 | 1.196 | 0.2800 | Yes |
| 28 | KIF5C |  |  | 240 | 1.170 | 0.2862 | Yes |
| 29 | HES6 |  |  | 250 | 1.150 | 0.2932 | Yes |
| 30 | BAZ2B |  |  | 280 | 1.071 | 0.2957 | Yes |
| 31 | GPC2 |  |  | 292 | 1.053 | 0.3016 | Yes |
| 32 | CDK2AP1 |  |  | 293 | 1.040 | 0.3096 | Yes |
| 33 | MEX3A |  |  | 296 | 1.038 | 0.3171 | Yes |
| 34 | CCSAP |  |  | 299 | 1.036 | 0.3246 | Yes |
| 35 | PCDH9 |  |  | 301 | 1.034 | 0.3323 | Yes |
| 36 | BICD1 |  |  | 314 | 1.020 | 0.3377 | Yes |
| 37 | GTF2I |  |  | 329 | 0.990 | 0.3426 | Yes |
| 38 | LZTS1 |  |  | 334 | 0.985 | 0.3493 | Yes |
| 39 | SOX4 |  |  | 377 | 0.928 | 0.3482 | Yes |
| 40 | ZEB1 |  |  | 385 | 0.920 | 0.3538 | Yes |
| 41 | KLHL24 |  |  | 419 | 0.885 | 0.3541 | Yes |
| 42 | SIPA1L2 |  |  | 421 | 0.882 | 0.3607 | Yes |
| 43 | SCARB2 |  |  | 426 | 0.876 | 0.3666 | Yes |
| 44 | CELSR2 |  |  | 428 | 0.875 | 0.3731 | Yes |
| 45 | PREX1 |  |  | 429 | 0.873 | 0.3797 | Yes |
| 46 | KDM5B |  |  | 434 | 0.868 | 0.3856 | Yes |
| 47 | DAAM1 |  |  | 444 | 0.860 | 0.3904 | Yes |
| 48 | CHRDL1 |  |  | 452 | 0.854 | 0.3955 | Yes |
| 49 | AGO1 |  |  | 453 | 0.853 | 0.4021 | Yes |
| 50 | RPAIN |  |  | 493 | 0.822 | 0.4007 | Yes |
| 51 | LDLRAD4 |  |  | 496 | 0.819 | 0.4066 | Yes |
| 52 | HDAC9 |  |  | 510 | 0.810 | 0.4102 | Yes |
| 53 | SCRN1 |  |  | 513 | 0.807 | 0.4160 | Yes |
| 54 | ZKSCAN1 |  |  | 524 | 0.794 | 0.4201 | Yes |
| 55 | TFDP2 |  |  | 527 | 0.789 | 0.4257 | Yes |
| 56 | ZNF493 |  |  | 543 | 0.773 | 0.4287 | Yes |
| 57 | AASS |  |  | 546 | 0.770 | 0.4342 | Yes |
| 58 | TRIM2 |  |  | 577 | 0.741 | 0.4340 | Yes |
| 59 | ENC1 |  |  | 590 | 0.734 | 0.4372 | Yes |
| 60 | GDAP1 |  |  | 617 | 0.715 | 0.4376 | Yes |
| 61 | ANKRD10 |  |  | 625 | 0.711 | 0.4417 | Yes |
| 62 | ARHGAP21 |  |  | 636 | 0.706 | 0.4451 | Yes |
| 63 | DDAH2 |  |  | 655 | 0.686 | 0.4468 | Yes |
| 64 | NCBP2 |  |  | 665 | 0.678 | 0.4502 | Yes |
| 65 | CALCOCO1 |  |  | 683 | 0.671 | 0.4520 | Yes |
| 66 | NREP |  |  | 701 | 0.660 | 0.4537 | Yes |
| 67 | IP6K2 |  |  | 722 | 0.647 | 0.4548 | Yes |
| 68 | HEG1 |  |  | 730 | 0.644 | 0.4583 | Yes |
| 69 | CDON |  |  | 764 | 0.622 | 0.4566 | Yes |
| 70 | PNISR |  |  | 769 | 0.621 | 0.4606 | Yes |
| 71 | SRGAP3 |  |  | 789 | 0.609 | 0.4615 | Yes |
| 72 | MIDN |  |  | 795 | 0.604 | 0.4652 | Yes |
| 73 | PRKX |  |  | 815 | 0.597 | 0.4660 | Yes |
| 74 | SEMA6D |  |  | 830 | 0.589 | 0.4678 | Yes |
| 75 | NFIC |  |  | 861 | 0.575 | 0.4663 | Yes |
| 76 | GTF2IRD2 |  |  | 878 | 0.569 | 0.4675 | Yes |
| 77 | PAG1 |  |  | 882 | 0.568 | 0.4712 | Yes |
| 78 | HIPK2 |  |  | 896 | 0.563 | 0.4730 | Yes |
| 79 | LRCH3 |  |  | 901 | 0.560 | 0.4765 | Yes |
| 80 | DANCR |  |  | 950 | 0.543 | 0.4713 | Yes |
| 81 | NUDT4 |  |  | 991 | 0.525 | 0.4674 | Yes |
| 82 | ZNF286A |  |  | 1020 | 0.514 | 0.4659 | Yes |
| 83 | CCDC88C |  |  | 1032 | 0.511 | 0.4676 | Yes |
| 84 | PAK3 |  |  | 1046 | 0.506 | 0.4690 | Yes |
| 85 | EVL |  |  | 1047 | 0.506 | 0.4728 | Yes |
| 86 | BMPR1A |  |  | 1074 | 0.501 | 0.4716 | Yes |
| 87 | SYNE2 |  |  | 1094 | 0.493 | 0.4716 | Yes |
| 88 | SORBS1 |  |  | 1099 | 0.492 | 0.4746 | Yes |
| 89 | CA12 |  |  | 1106 | 0.490 | 0.4772 | Yes |
| 90 | BSDC1 |  |  | 1139 | 0.483 | 0.4746 | Yes |
| 91 | ZNF292 |  |  | 1153 | 0.479 | 0.4757 | Yes |
| 92 | VASH1 |  |  | 1165 | 0.476 | 0.4772 | Yes |
| 93 | TP53INP1 |  |  | 1174 | 0.473 | 0.4792 | Yes |
| 94 | NEUROD1 |  |  | 1180 | 0.471 | 0.4819 | Yes |
| 95 | AUTS2 |  |  | 1211 | 0.462 | 0.4795 | Yes |
| 96 | WDR6 |  |  | 1216 | 0.459 | 0.4822 | Yes |
| 97 | SYNRG |  |  | 1226 | 0.457 | 0.4840 | Yes |
| 98 | SHISA5 |  |  | 1237 | 0.455 | 0.4855 | Yes |
| 99 | SOGA1 |  |  | 1242 | 0.453 | 0.4882 | Yes |
| 100 | PTPRS |  |  | 1253 | 0.451 | 0.4897 | Yes |
| 101 | CPEB2 |  |  | 1256 | 0.451 | 0.4927 | Yes |
| 102 | VANGL2 |  |  | 1262 | 0.450 | 0.4952 | Yes |
| 103 | PAX6 |  |  | 1275 | 0.445 | 0.4962 | Yes |
| 104 | PPP1R17 |  |  | 1286 | 0.442 | 0.4976 | Yes |
| 105 | SLC4A7 |  |  | 1292 | 0.441 | 0.5000 | Yes |
| 106 | ATP6V0A1 |  |  | 1320 | 0.436 | 0.4981 | Yes |
| 107 | EPHA3 |  |  | 1362 | 0.425 | 0.4933 | Yes |
| 108 | FOXN2 |  |  | 1381 | 0.421 | 0.4930 | Yes |
| 109 | RASGRP1 |  |  | 1384 | 0.420 | 0.4958 | Yes |
| 110 | CYTH1 |  |  | 1391 | 0.419 | 0.4979 | Yes |
| 111 | PABPN1 |  |  | 1396 | 0.417 | 0.5003 | Yes |
| 112 | CSNK1E |  |  | 1412 | 0.413 | 0.5005 | Yes |
| 113 | TMTC2 |  |  | 1416 | 0.412 | 0.5030 | Yes |
| 114 | SOX5 |  |  | 1422 | 0.412 | 0.5052 | Yes |
| 115 | MAPK10 |  |  | 1471 | 0.398 | 0.4989 | No |
| 116 | FYN |  |  | 1483 | 0.394 | 0.4997 | No |
| 117 | PPFIA2 |  |  | 1516 | 0.385 | 0.4964 | No |
| 118 | ZNF302 |  |  | 1520 | 0.385 | 0.4987 | No |
| 119 | SPATA13 |  |  | 1530 | 0.382 | 0.4999 | No |
| 120 | ZNF562 |  |  | 1533 | 0.382 | 0.5024 | No |
| 121 | ZNF638 |  |  | 1559 | 0.375 | 0.5004 | No |
| 122 | ZNF3 |  |  | 1567 | 0.374 | 0.5019 | No |
| 123 | PCMTD1 |  |  | 1568 | 0.374 | 0.5047 | No |
| 124 | TMEM158 |  |  | 1588 | 0.369 | 0.5038 | No |
| 125 | NOL4L |  |  | 1642 | 0.359 | 0.4962 | No |
| 126 | PTPN4 |  |  | 1682 | 0.353 | 0.4913 | No |
| 127 | MARCKS |  |  | 1713 | 0.348 | 0.4881 | No |
| 128 | FBXL20 |  |  | 1732 | 0.343 | 0.4872 | No |
| 129 | NCSTN |  |  | 1744 | 0.341 | 0.4876 | No |
| 130 | HECTD4 |  |  | 1764 | 0.337 | 0.4865 | No |
| 131 | MKLN1 |  |  | 1769 | 0.336 | 0.4883 | No |
| 132 | BRWD1 |  |  | 1822 | 0.328 | 0.4806 | No |
| 133 | SPAG9 |  |  | 1836 | 0.326 | 0.4805 | No |
| 134 | ASIC1 |  |  | 1838 | 0.326 | 0.4828 | No |
| 135 | KLF13 |  |  | 1871 | 0.318 | 0.4790 | No |
| 136 | PLCE1 |  |  | 1895 | 0.315 | 0.4769 | No |
| 137 | ELAVL2 |  |  | 1938 | 0.307 | 0.4710 | No |
| 138 | ELMO1 |  |  | 1966 | 0.303 | 0.4681 | No |
| 139 | NIPBL |  |  | 1997 | 0.297 | 0.4645 | No |
| 140 | GLCE |  |  | 2028 | 0.292 | 0.4608 | No |
| 141 | SNCAIP |  |  | 2043 | 0.290 | 0.4603 | No |
| 142 | SEZ6L |  |  | 2065 | 0.287 | 0.4584 | No |
| 143 | NRN1 |  |  | 2123 | 0.281 | 0.4494 | No |
| 144 | ATP6V1G1 |  |  | 2148 | 0.277 | 0.4468 | No |
| 145 | SMIM8 |  |  | 2162 | 0.274 | 0.4464 | No |
| 146 | TRIM33 |  |  | 2196 | 0.268 | 0.4419 | No |
| 147 | MYT1 |  |  | 2292 | 0.257 | 0.4253 | No |
| 148 | RPL8 |  |  | 2389 | -0.257 | 0.4085 | No |
| 149 | ZMIZ1 |  |  | 2414 | -0.259 | 0.4058 | No |
| 150 | SSR4 |  |  | 2425 | -0.260 | 0.4058 | No |
| 151 | CUL1 |  |  | 2617 | -0.285 | 0.3706 | No |
| 152 | FOXP4 |  |  | 3120 | -0.349 | 0.2751 | No |
| 153 | SPIRE1 |  |  | 3141 | -0.352 | 0.2739 | No |
| 154 | IVNS1ABP |  |  | 3154 | -0.353 | 0.2742 | No |
| 155 | ANK3 |  |  | 3216 | -0.363 | 0.2651 | No |
| 156 | SVIL |  |  | 3218 | -0.363 | 0.2676 | No |
| 157 | RNF144A |  |  | 3276 | -0.371 | 0.2593 | No |
| 158 | MAP7D1 |  |  | 3281 | -0.372 | 0.2614 | No |
| 159 | PGRMC2 |  |  | 3416 | -0.393 | 0.2382 | No |
| 160 | CORO1C |  |  | 3478 | -0.405 | 0.2293 | No |
| 161 | ACTB |  |  | 3512 | -0.412 | 0.2260 | No |
| 162 | TSPAN5 |  |  | 3515 | -0.412 | 0.2288 | No |
| 163 | NRG1 |  |  | 3935 | -0.494 | 0.1506 | No |
| 164 | SPATS2 |  |  | 4033 | -0.521 | 0.1356 | No |
| 165 | PSMB7 |  |  | 4101 | -0.541 | 0.1266 | No |
| 166 | FLRT3 |  |  | 4285 | -0.593 | 0.0954 | No |
| 167 | CACNA2D1 |  |  | 4302 | -0.598 | 0.0968 | No |
| 168 | RND3 |  |  | 4402 | -0.635 | 0.0823 | No |
| 169 | FMNL2 |  |  | 4489 | -0.673 | 0.0706 | No |
| 170 | EPHB2 |  |  | 4657 | -0.768 | 0.0438 | No |
| 171 | EZR |  |  | 4714 | -0.803 | 0.0390 | No |
| 172 | RANBP1 |  |  | 4741 | -0.823 | 0.0402 | No |
| 173 | PRDX1 |  |  | 4783 | -0.848 | 0.0386 | No |
| 174 | CLMP |  |  | 5009 | -1.111 | 0.0031 | No |
| 175 | COTL1 |  |  | 5019 | -1.125 | 0.0099 | No |
| 176 | MFAP2 |  |  | 5087 | -1.259 | 0.0064 | No |
| 177 | SORBS2 |  |  | 5124 | -1.342 | 0.0096 | No |
| 178 | NR2F1 |  |  | 5135 | -1.386 | 0.0183 | No |
| 179 | NRP1 |  |  | 5177 | -1.553 | 0.0221 | No |
Table: GSEA details [plain text format]

  

Fig 2: RICHARDS\_NOWAKOWSKI\_SCIENCE\_2017\_IPC\_NEN1\_UPREG: Random ES distribution      
 Gene set null distribution of ES for **RICHARDS\_NOWAKOWSKI\_SCIENCE\_2017\_IPC\_NEN1\_UPREG**
